# Twist1 promotes radioresistance in nasopharyngeal carcinoma

**DOI:** 10.18632/oncotarget.12875

**Published:** 2016-10-25

**Authors:** Linli Zhang, Beibei Su, Wei Sun, Wenwen Li, Min Luo, Dongbo Liu, Qi Mei, Guoxian Long, Guangyuan Hu, Guoqing Hu

**Affiliations:** ^1^ Department of Oncology, Tongji Hospital, Tongji Medical College, Huazhong University of Science and Technology, Wuhan, Hubei, 430030, P.R. China

**Keywords:** Twist1, radioresistance, nasopharyngeal carcinoma, DNA damage repair, apoptosis

## Abstract

With the development of advanced imaging and radiation technologies, radiotherapy has been employed as the principal treatment approach for nasopharyngeal carcinoma (NPC). So far, a number of patients still suffer from the failure of this treatment due to the acquired radioresistance, but the underlying mechanisms are still poorly defined. In this study, we found that Twist1, participating in a variety of cell biological process, was associated with the malignancy of NPC and could induce NPC radioresistance *in vitro* and *in vivo*. Mechanically, Twist1 could promote the accumulation of DNA damage repair and inhibit the apoptosis of NPC cells. Therefore, our study not only elucidates the significant role of Twist1 in radioresistance of NPC, but also highlights Twist1 as a potential therapeutic target for NPC.

## INTRODUCTION

Nasopharyngeal carcinoma (NPC), arising from the mucosal epithelium of the nasopharynx, is a locally malignant cancer with high metastatic potential [1]. NPC frequently appears in Southeast Asia and Africa, but it is relatively uncommon in most other countries [2]. Radiotherapy remains the most powerful treatment modality for NPC, especially with the development of advanced imaging and radiation technologies [3]. However, radioresistance is still a major hurdle to treatment success in some cases of NPC. The exact mechanisms underlying radioresistance of NPC remain unknown [4].

Twist1, a highly conserved basic helix-loop-helix transcription factor, regulates embryonic morphogenesis, cell migration, and differentiation of mesodermal, myoblast, and osteoblast. Recently, Twist1 has been found to be a vital oncogene that promotes the development and progression of malignant tumor by enhancing tumor-related functions such as epithelial-to-mesenchymal transition (EMT), angiogenesis, degeneration of ECM, and anti-apoptosis [5, 6]. In NPC, Twist1 has also been suggested to contribute to metastasis, malignant progression [7] and drug resistance (Taxol) of tumor [8]. Recently, increasing studies have indicated that EMT is associated with radioresistance [9]. Since Twist1 is an EMT regulator and plays a key role in the development of NPC, Twist1 may be a potential factor to be related to radioresistance. However, it is still unclear whether Twist1 could induce radioresistance of NPC.

Biologically, to safeguard genomic integrity, cells generate an acute and coordinated reaction in response to increased DNA damage from radiation, which is known as DNA damage response (DDR). DDR is a complex pathway that includes sense of damage, transduction of damage and cellular response to DNA damage [10]. DDR contributes to radioresistance in cancer cells [11]. Furthermore, Radiation-induced apoptosis, which can be regulated by the interactions between anti-apoptotic proteins and pro-apoptotic proteins, is also considered to be one of the main causes of cell death following exposure to irradiation [12]. Reducing of apoptosis can promote the radioresistance of cancer cells. Thus, here we reported that Twist1 induced radioresistance in NPC by increasing the DNA damage repair and inhibiting the apoptosis, which emphasized the potential therapeutic role of Twist1 for NPC patients with radioresistane.

## RESULTS

### Twist1 is correlated with malignance and radioresistance of NPC

**Figure 1:**
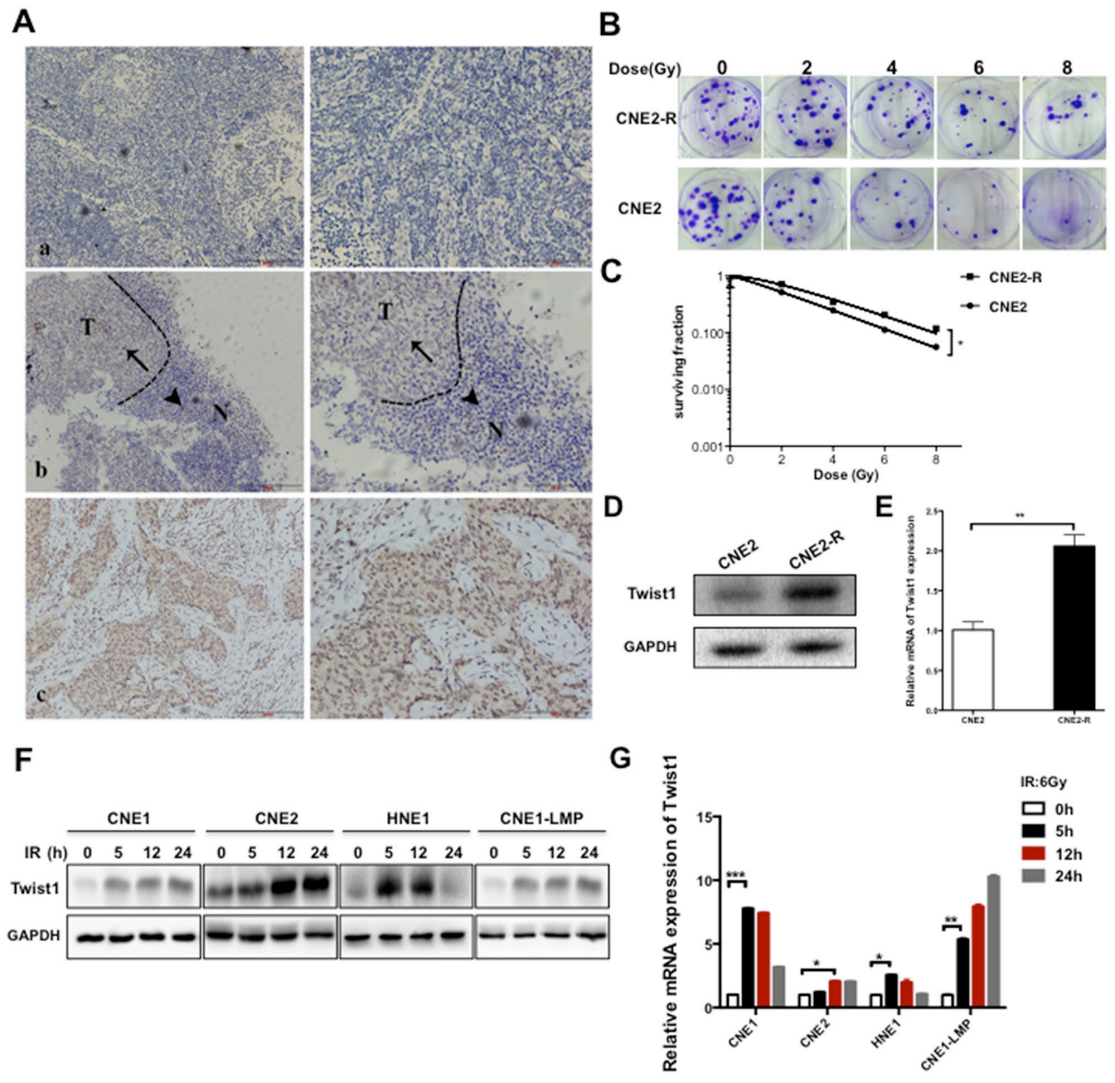
Twist1 is correlated with malignance and radioresistance of NPC **A.** The representative immunohistochemical (IHC) stainings for Twist1 in paraffin-embedded tissue sections. a, negative stainings of Twist1 in nasopharyngeal inflammation tissues. b, the different expressions of Twist1 in normal (N) and tumorous (T) tissues, as indicated by arrows. c, positive stainings of Twist1 in nasopharyngeal cancer. Photos were taken under 10X, 200X magnification respectively. **B.** The representative colony formation images of radiation resistant CNE2 cells (CNE2-R) and CNE2 cells, survival curves were prepared in **C.** the error bars represent means ± standard deviations (SDs) from 3 independent experiments, *P<0.05 versus (vs.) control. **D.** The expressions of Twist1 were showed by immunoblot (IB) in CNE2 and CNE2-R. **E.** The quantification of Twist1 mRNA in CNE2 and CNE2-R cells, the error bars are means ± SDs from 3 independent experiments, **P<0.01 vs. control. **F.** The IB analysis of WCLs (whole cell lysates) derived from different NPC cell lines after exposure to radiation specific for Twist1. **G.** The quantification and analysis of Twist1 mRNA in different NPC cell lines irradiated with 6 Gy radiation, the error bars are means ± SDs from 3 independent experiments, *P<0.05 vs. control;**P<0.01 vs. control; ***P<0.005 vs. control.

Twist1 was reported to play a pivotal role in malignant progression and metastasis of tumor such as blood cancer, colorectal cancer and lung cancer [13-15]. However, its oncogenic role is not well evaluated in NPC. To this end, we examined the expressions of Twist1 in NPC samples via immunohistochemistry (IHC) approach and observed that the cancerous tissues displayed remarkably higher levels of Twist1 relative to the adjacent normal tissues (Figure 1A). In detail, high expression of Twist1 was found in 63% (14 of 22) of NPC patients, in which 77.8% of patients with lymph node metastasis and 63% of patients with distance metastasis (Table 1). Consistent with these findings, aberrantly higher expression of Twist1 was also associated with advanced clinical stage and T classification of NPC patients (Table 1). Taken together, these data indicated that Twist1 might paly a critical role in the progression of NPC.

**Table 1:** Association between Twist1 and clinicopathological features of nasopharyngeal cancer patients

Characteristics		Twist				P-value
		−		+		
		No.	%	No.	%	
Age(years)	<=46	4	50	4	50	0.386
	>46	4	28.6	10	71.4	
Gender	Male	4	25	12	75	0.137
	Female	4	66.7	2	33.3	
Clinical stage	I-II	5	83.3	1	16.7	0.011
	III-IV	3	18.8	13	81.3	
Lymph node metastasis	Negative	4	100	0	0	0.01
	Positive	4	22.2	14	77.8	
T classification	T1	1	100	0	0	0.011
	T2	5	83.3	1	16.7	
	T3	1	20	4	80	
	T4	1	10	9	90	
Distance metastasis	Negative	8	53.3	7	46.7	0.022
	Positive	0	0	7	100	
WHO histological classification	NKUC	2	66.7	1	33.3	0.527
	NKDC	6	31.6	13	68.4	

Abbreviations: NKDC, nonkeratinizing differentiated carcinoma; NKUC, nonkerat- -inizing undifferentiated carcinoma.

To uncover the mechanism of NPC radioresistance, we generated the acquired radioresistant CNE2 (CNE2-R) cell line by the treatment of progressively-increasing radiation (Figure 1B-1C), and observed that the both protein and mRNA levels of Twist1 were elevated in CNE2-R cells (Figure 1D-1E). Similar results were obtained in different NPC cell lines including CNE1, CNE2, HNE1 and CNE1-LMP after exposure to 6 Gy radiation (Figure 1F-1G). Together, These data coherently supported the notion that Twsit1 could be induced by radiation treatment in NPC.

### Twist1 induces radioresistance in NPC cells

**Figure 2:**
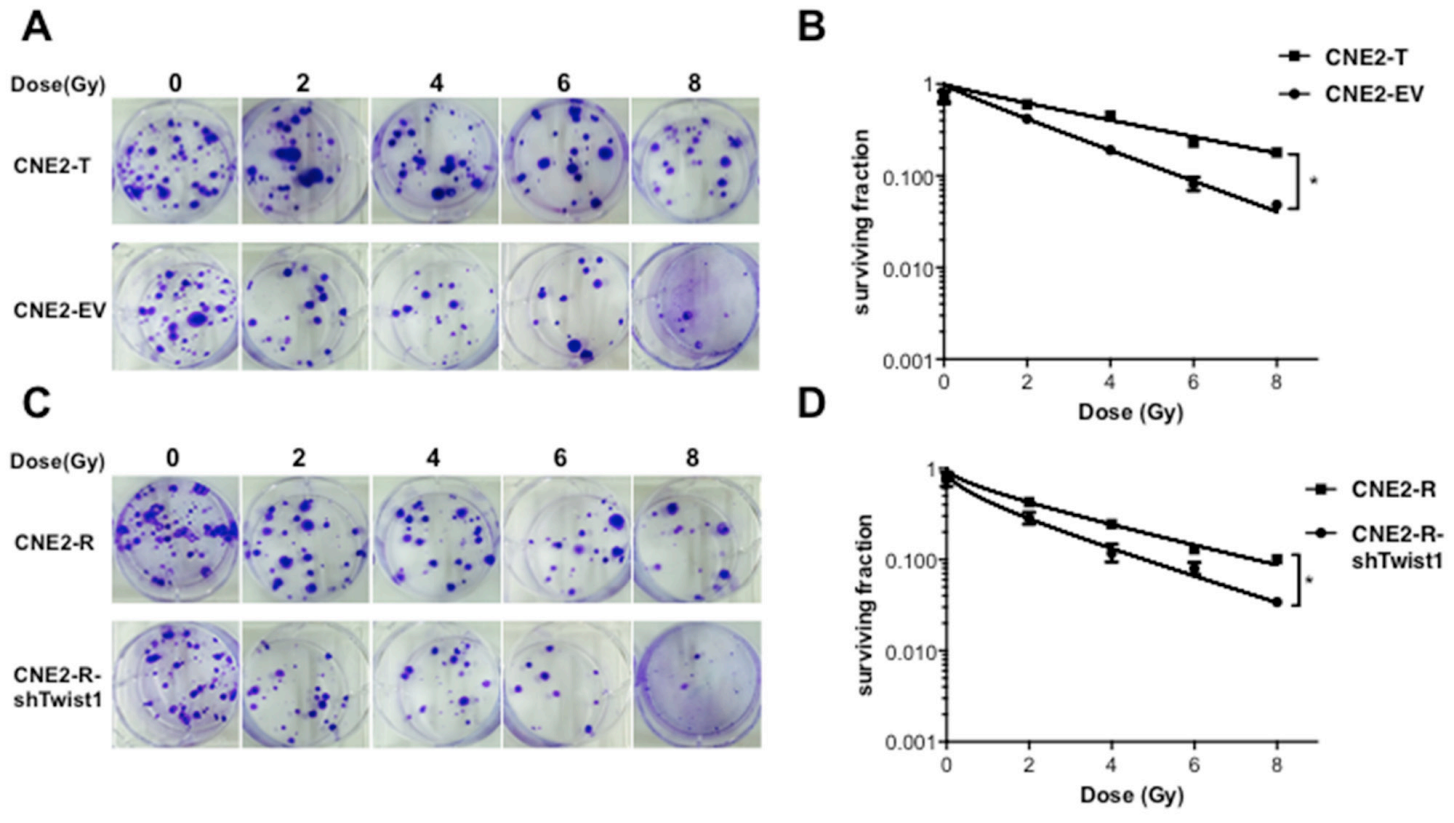
Twist1 induces radiation resistance in NPC cells **A.** The representative images of colony formation in Twist1 overexpressed CNE2 cells (CNE2-T) and control cells (CNE2-EV) irradiated with indicated radiation, survival curves were prepared in **B.** the error bars are means ± SDs from 3 independent experiments, *P<0.05 vs. control. **C.** The representative images of colony formation in Twist1 knocking-down CNE2-R cells (CNE2-R-shTwist1) and control cells (CNE2) irradiated with indicated doses of radiation, survival curves were prepared in **D.** the error bars are means ± SDs from 3 independent experiments, *P<0.05 vs. control.

To further investigate the potential role of Twist1 in radioresistance of NPC, we engineeringly generated Twist1 stably expressing CNE2 cell line (CNE2-T) and Twist1 knockdown CNE2-R cell line (CNE2-R-shTwist1) (Supplementary Figure S1, B-C). We further exposed these engineered cell lines to different doses of radiation, and found that CNE2-T cells had relatively higher colony survival compared to CNE2-EV cells after irradiation (Figure 2A-2B). Consistent with this result, CNE2-R-shTwist1 cells showed lower colony survival relative to CNE2-R cells (Figure 2C-2D). Together, These data supported the notion that Twist1 might induce radioresistance of NPC cells.

### Twist1 enhances DNA damage repair in NPC cells

**Figure 3:**
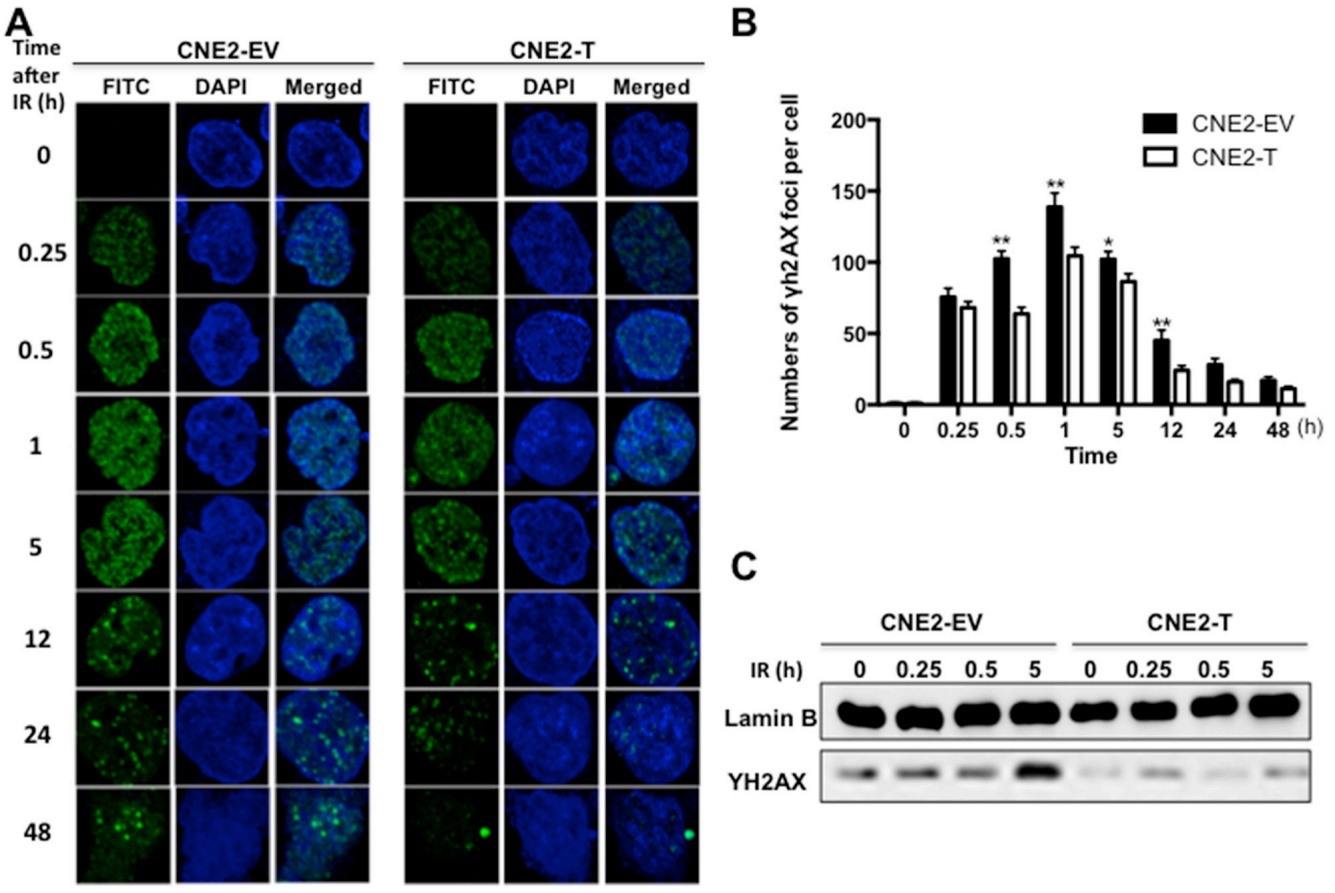
Twist1 accumulates DNA damage repair in NPC cells **A.** The immunostainings of CNE2-EV and CNE2-T cells after exposed to radiation (6Gy) for the indicated time, FITC: γ-H2AX foci, DAPI: Nuclear. The foci for each time point were counted in 3 independent experiments (50 nuclei each) and analyzed in **B.** a student's t-test was used to evaluate the statistical significance. The error bars are means ± SDs from 3 independent experiments, *P<0.05 vs. control, **P<0.01 vs. control. **C.** The IB analysis of nuclear extracts derived from CNE2-EV and CNE2-T cells exposed to radiation (6Gy) for the indicated time.

To better understand the mechanisms underlying Twist1-induced NPC radioresistance, we tested the expressions of γH2AX, a major effector to DNA damage [16], in NPC cells after irradiation. As shown in Figure 3A, the number of γH2AX foci significantly increased in CNE2-EV and CNE2-T cells after treatment of 6 Gy radiation and reached a peak at 1 hour. However, the number of γH2AX foci in CNE2-EV cells was observably higher than the number in CNE2-T cells at different time point. Moreover, the rest of the foci were almost eliminated in the CNE2-T cells after irradiated for 12 hours, while the foci still existed at 48 hours after irradiation in CNE2-EV cells (Figure 3A-3B). Consistent with this data, the immunoblotting results also showed that the protein levels of γH2AX in CNE2-EV cells were higher than the levels in CNE2-T cells at indicated time point (Figure 3C). These results together suggested that Twist1 compromised DNA damage accumulation after treatment of radiation.

### Twist1 inhibits apoptosis of NPC cells after exposed to radiation

**Figure 4:**
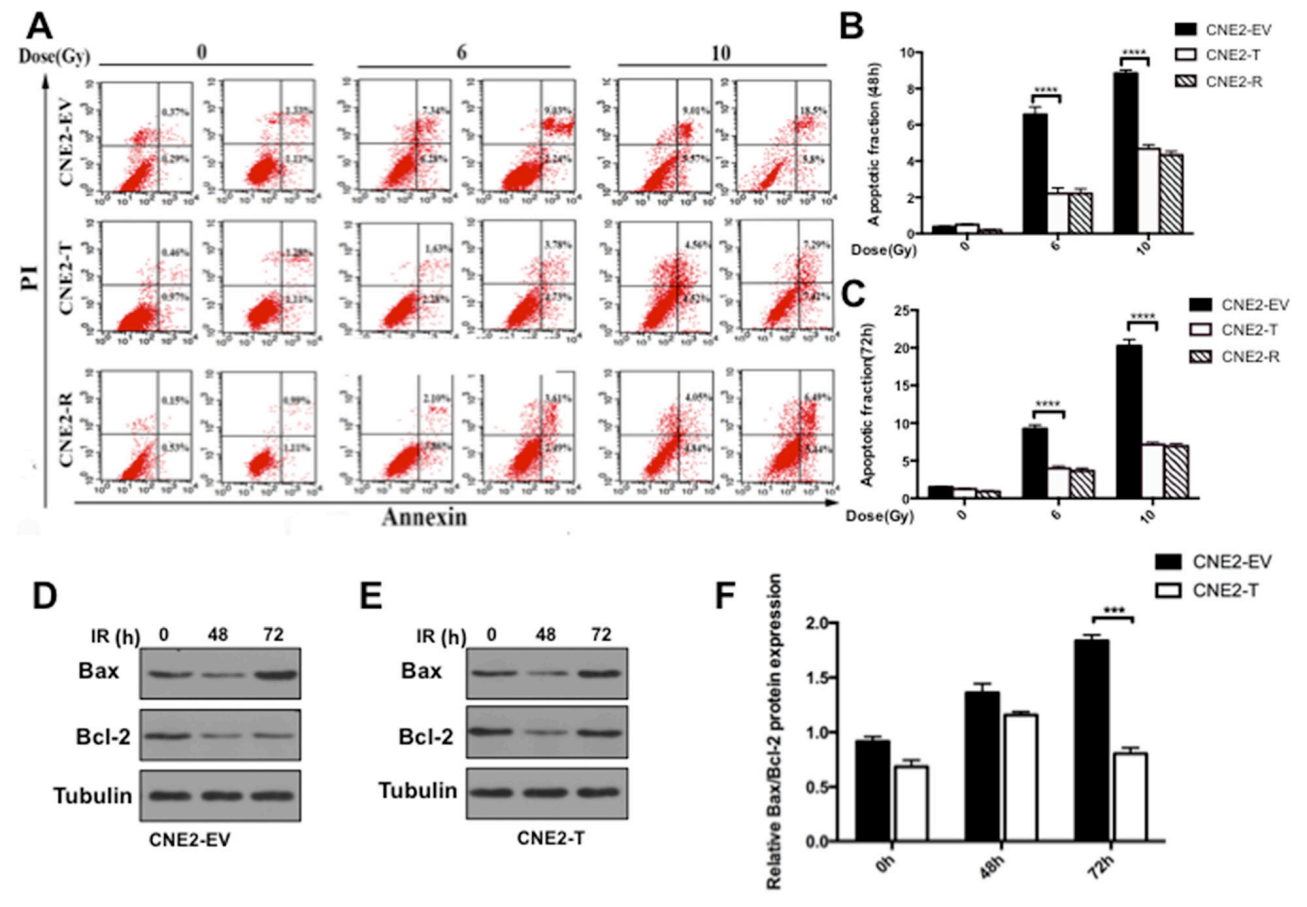
Twist1 impairs radiation induced apoptosis in NPC cells **A.** The representative flow cytometry images of cells treated with radiation of 6 Gy and 10 Gy for 24 hours and 72 hours. **B.** and **C.** Quantifications of cell apoptosis at 48h and 72h respectively. data are means ± SDs from 3 independent experiments, ****P<0.001 vs. control. **D.** and **E.** The IB analysis of WCLs from CNE2-EV and CNE2-T cells after treated with 6 Gy radiation for 48 hours and 72 hours specific for Bcl-2 and Bax. Quantification and analysis of relative Bcl-2/Bax rations was showed in **F.** the error bars are means ± SDs from 3 independent experiments, ****P<0.001 vs. control.

Apoptosis is the major response of cancer cells to radiation therapy [12]. To further test whether Twist1 induces radioresisitance via the inhibition of apoptosis, we examined the apoptosis of CNE2 cells after radiation treatment. To this end, we observed that as reported previously, radiation enhanced cells apoptosis in a dose-dependent manner [12], while enforcing expression of Twist1 significantly decreased CNE2 cells apoptosis compared with that of CNE2-EV cells (Figure 4A-4C). As we know, apoptosis is majorly governed by the balance of anti-apoptotic proteins including Bcl2, Bcl-xL and pro-apoptotic proteins such as Bax, Bak [17]. Here, we found Twist1 could decrease the levels of Bax/Bcl-2 in CNE2 cells after exposure to 6Gy radiation over 72 hours (Figure 4D-4F), indicated that Twist1 induced radioresistance in large part due to restraining NPC cells apoptosis by altering Bax/Bcl-2.

### Twist1 compels cells resistant to radiation in xenograft model

**Figure 5:**
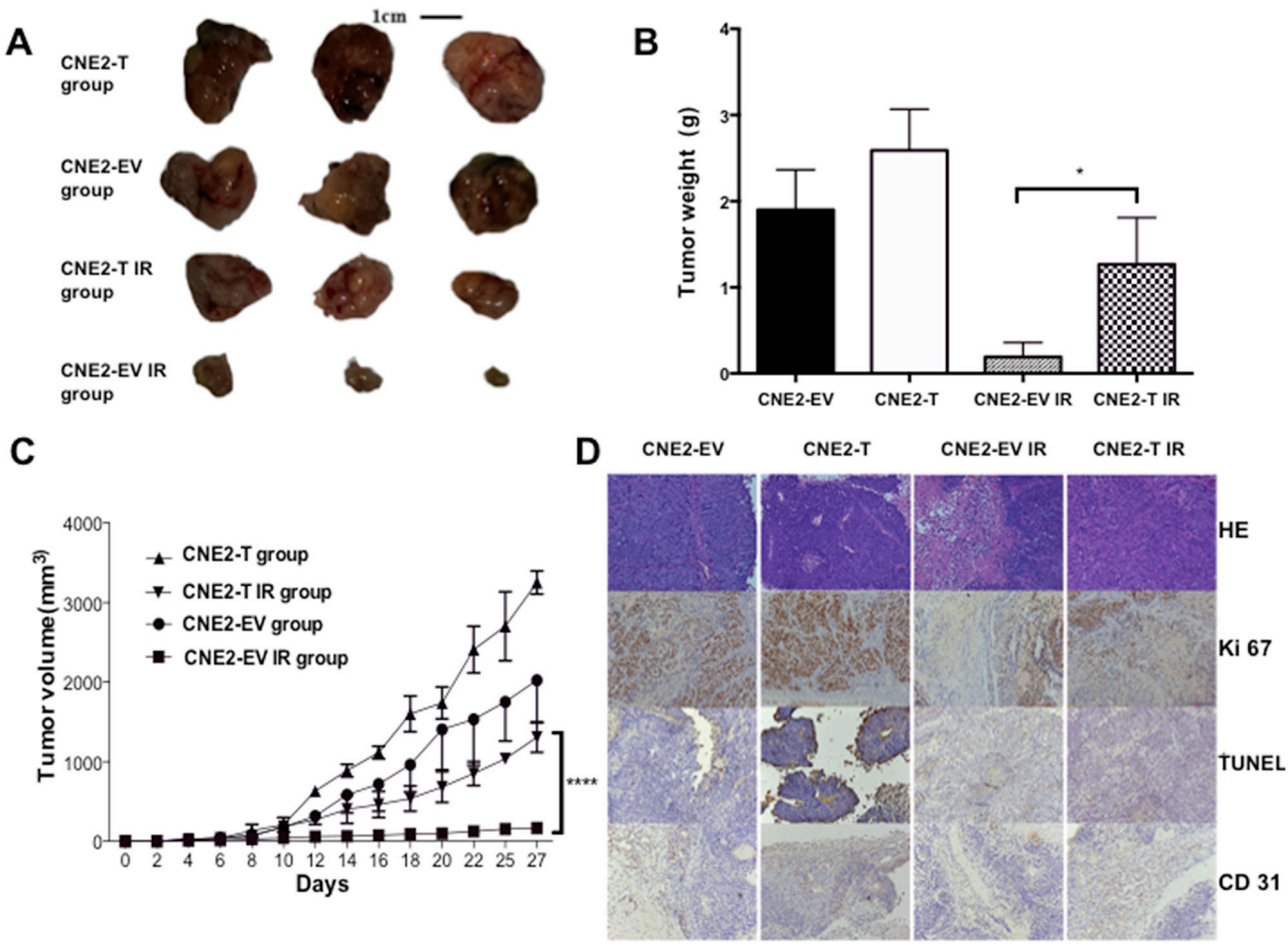
Twist1 promotes tumor growth in Xenograft model **A.** Tumor volumes from each group were tracked for 4 weeks, the representative tumor samples from each group were shown. quantifications were showed in **B.** the error bars are means ± SDs from 3 independent experiments, *P<0.05 vs. control. **C.** Tumor volumes from each group were followed for 4 weeks, the data are means ± SDs from 3 independent experiments, ****P<0.001 vs. control. **D.** Representative immunohistochemical stainings of hematoxylin-eosin (HE), TUNEL, Ki-67 and CD31 from tumor samples in each group were shown. (magnification: 200X).

To further validate whether Twist1 could promote NPC radioresistance *in vivo*, we employed a xenograft mouse model, where we injected CNE2-EV and CNE2-T cells to nude mice, and found the tumor sizes of the CNE2-T group were larger than those in the CNE2-EV group. Furthermore, the tumors in the CNE2-T irradiation (IR) group were much lager than those in the CNE2-EV IR group after exposure to radiation, (Figure 5A-5B). Consistent with these data, Tumors in CNE2-T group also grew faster than CNE2-EV group (Figure 5C). Furthermore, decreases of Ki-67 signals and microvessel density measured by CD31 staining, and increases in apoptosis signal detected by TUNEL assays were observed in the radiation treatment groups. More strikingly, the phenotypes mentioned above were most significant in the CNE2-EV IR group relative to CNE2-T IR group (Figure 5D). These data together implied that Twist1 enhanced radioresistance in vivo might through acceleration of the cell proliferation, angiogenesis and the inhibition of apoptosis.

## DISCUSSION

Twist1 has a wide variety of biological functions, including enhancement of the motility, invasiveness and vasculogenic mimicry formation in cancer cells [5, 18]. Previous studies suggested that Twist1 was a novel factor in the development and progression of many cancers, such as prostate cancer [19]. In this study, we found that Twist1 was expressed in nasopharyngeal carcinoma tissues but not in the adjacent normal tissues (Figure 1A). Furthermore, increased Twist1 protein levels were positively associated with the advanced clinical stage and metastasis (Table 1). Our data are in line with previous studies that highlight the contribution of Twist1 in the development and metastasis of NPC. Interestingly, we found Twist1 was accumulated in radioresistant NPC cells and further observed that radiation could induce Twist1 expression both in mRNA and protein levels (Figure 1D, 1E). Overexpression of Twist1 impelled NPC cells resistant to radiation, while deletion of Twist1 sensitized NPC cells to radiation therapy both in cell and *in vivo* (Figure 2A, 2C; Figure 5A). These implied that Twist1 plays a pivotal role in radioresistance of NPC, and Twist1 may be a potential target in radiation-resistant NPC.

DDR, a sophisticated network of DNA damage-response, was reported to be one of the mechanisms responsible for radioresistance in cancer cells [11]. It involves in an elaborate signaling network that connects sense of damage, control of cell cycle, different types of DNA repair, and other physiological reactions in cells [20]. Phosphorylation of H2AX (γH2AX) is a crucial process in the DDR and plays a role in initiating the repair of DNA double-strand breaks (DSBs) [21]. Fewer γH2AX foci indicate a more efficient repair of DSBs and an increase of radioresistance [16]. In the present study, we demonstrated that upregulation of Twist1 decreased the expression of γH2AX (Figure 3A), which implied the acceleration of DSBs repair and higher radioresistance. Furthermore, if DNA damage repairs after irradiation are failed, the cells are driven to apoptosis. If repairs are successful, the cells endure proliferation [22]. We observed that Twist1 retrained radiation-induced apoptosis (Figure 4A). In the meanwhile, Twist1 also up-regulated anti-apoptotic protein Bcl-2 and down-regulated pro-apoptotic protein Bax after cells exposure to radiation (Figure 4E).

Ultimately, our findings indicate that Twist1 may induce radioresistance in NPC cells. The molecular mechanism governing this radioresistance may be effects of Twist1 on promotion of DDR and decreasing of apoptosis. Taken in combination with previous findings, the present study suggests that Twist1 may be a critical oncogene of NPC and may be a potential target in condition of radiation-resistant NPC. Thus, the role of Twist1 in radioresistance of NPC deserves further attention.

## MATERIALS AND METHODS

### Tissue samples

Tumor tissue samples were collected from 22 nasopharyngeal cancer patients, while non-cancerous nasopharyngeal samples were collected from 20 nasal polyps patients who had undergone primary biopsies at Tongji Hospital of Tongji Medical College, Huazhong University of Science and Technology between June 2013 and November 2014. Clinical stages were classified according to the seventh edition of the AJCC Cancer Staging Manual. None of the patients received therapy prior to biopsy.

### Tissue immunohistochemistry

Tissue samples embedded in paraffin were cut into 5μm-thick sections and the signal of Slides was generated with indicated antibodies and DAB following the protocols. Apoptosis of tissue was detected by terminal deoxynucleotidyl transferase-meditated dUTP-fluorescein nick end-labeling (TUNEL) using the In Situ Cell Death Detection Kit, POD (Roche, Basel, Switzerland) according to the manufacturer's instructions.

### Cell lines and lentivirus vectors

The human NPC cell lines HNE1, CNE1, CNE1-LMP1, and CNE2 were obtained from the Cancer Research Institute of Central South University (Changsha, China). The CNE2-radioresistance (CNE2-R) cell line was generated from a poorly-differentiated CNE2 cell line by exposing to progressively-increasing radiation over the course of 6 months. All cell lines were maintained in RPMI 1640 (Hyclone) and supplemented with 10% fetal bovine serum (Sijiqing) at 37°C in 5% CO_2_. The Twist1-plasmid lentiviral particle, Sh-Twist1 lentiviral particle and the empty-vector lentiviral particle used as controls were purchased from GeneChem (Shanghai, China). Lentiviruses were transfected into CNE2 cells following the instructions of the manufacturer. In addition, 3 mg/mL Puromycin (Sigma) was used to sort out stable transfected cell lines.

### X irradiation (radiation exposure)

X irradiation was performed using the RS2000 X-ray Biological Research Irradiator (3mm copper filter, 160kV, 25mA; Rad Source Technologies, GA, USA).

### Western blotting, antibodies

Following treatment, nucleoproteins and cytoplasmic proteins were extracted using Nuclear and Cytoplasmic Extraction Reagents (Promoter Biotech) following the instructions of the manufacturer. The protein concentrations of lysates were measured by the Synergy™ H1 (Biotek) using the Beyotime protein assay reagent. Same amounts of whole cell lysates were resolved by SDS-PAGE and immunoblotted with indicated antibodies. The Micro-Chemi (DNR Bio-Imaging Systems) and ImageJ software was used for signal detection and quantification. Anti-Twist1, horseradish peroxidase-conjugated goat anti-mouse secondary antibodies were purchased from Abcam. Anti-E-cadherin, Anti-N-cadherin, Anti-Vimentin, anti-Bax, anti-Bcl-2, anti-pH2AX (Ser139) (γH2AX), anti-Lamin B were purchased from Cell Signaling Technology. Anti-GAPDH and the horseradish peroxidase-conjugated goat anti-rabbit secondary antibodies were purchased from Epitomics.

### RNA extraction, cDNA synthesis, and real-time PCR

All RNA was extracted from the cells using RNAiso Plus (Takara) following the manufacturer's instructions. Then the first strand of cDNA was reversed using the PrimeScriptTM 1st Strand cDNA Synthesis Kit (Takara), again according to the manufacturer's instructions. The qRT-PCR was run in the 7900HT Fast Real-Time PCR System (Applied Biosystem, USA) and detected by using SYBR Select Master (Life Technologies). A melting curve was constructed for each primer pair to confirm amplification product specificity. The PCR products were verified using 2% agarose gel electrophoresis and analyzed using the Gel Doc™ XR Imaging System (Bio-Rad). The primers for PCR were designed using the Primer Premier 5.0 software (Premier Biosoft International) and are as listed:

Twist1 Forwards-GTCCGCAGTCTTACGAGGAG;
Twist1 Reverse-GCTTGAGGGTCTGAATCTTGCT;
GAPDH Forwards-GGTCGGAGTCAACGGATTTG;
GAPDH Reverse-GGAAGATGGTGATGGGATTTC.

### Immunofluorescence assay

The cells were seeded at same cell density in slides, then they were incubated at 37°C for 4-10 h for attachment and irradiated with 6 Gy of X-ray respectively. Immunofluorescence was stained by using Alexa488 (Life Technologies) at indicated time point according to the instructions of the manufacturer. Images were taken using the FluoView™ FV1000 Confocal Laser Scanning Biological Microscope (Olympus, Tokyo, Japan).

### Colony formation assay

The cells were seeded at same cell density in 60mm cell culture dishes. Then they were incubated at 37°C for 4-10 h for attachment and irradiated with 0, 2, 4, 6, 8, or 10 Gy of X-ray respectively. Following this, 10-14 days of incubation were allowed for colony formation. The cells were fixed with methanol and then stained with 0.1% crystal violet. Colonies of at least 50 cells were counted. The surviving fraction (SF) was calculated as the number of colonies / (number of cells seeded × plating efficiency), where plating efficiency (PE) was defined as the number of control colonies obtained/number of control cells seeded.

### Apoptosis analysis

The cells were then exposed to 6 Gy and 10 Gy radiation and harvested after incubation for 48 h or 72 h. In order to perform apoptosis analysis, cells were stained with AnnexinV-fluorescein isothiocyanate (FITC) and PI using FITC Apoptosis Kit (Nanjing KeyGen Biotech) following the manufacturer. The apoptotic cells were determined by flow cytometry using a FACSort (BD Biosciences) and analyzed using CellQuest software.

### Transwell assay

Transwell 24-well plates with 8-μm diameter filters (Corning) were used to detect the migration and invasion abilities of cells. Approximately 1×10^5^ cells in 200 μl of a serum-free medium were placed in the upper chamber and 500 μl 10% fetal serum was placed in the lower chamber. After incubation for 20-24h, the cells on the underside of the filters were examined and counted under a microscope at 200X magnification.

### In vivo xenograft study

Female BALB/c nude mice (4 weeks old; HFK Bio-Technology) were used in this study. In brief, 1.5× 10^6^ cells in 0.1 ml serum-free medium were injected subcutaneously into the right posterior legs of the mice. The animals were randomly divided into 4 groups of 6 animals each: the untreated CNE2-EV group, the untreated CNE2-T group, the CNE2-EV irradiation (CNE2-EV IR) group, and the CNE2-T irradiation (CNE2-T IR) group. Tumors were measured every other day, and volume was calculated using the formula ([length]×[width]^2^)/2. When the tumors reached 50 mm^3^ in volume, the tumor areas in 12 mice were irradiated with 10 Gy X-rays and tracked for approximately 4 weeks. The experiments were then terminated.

### Statistical analysis

All experiments were repeated independently at least 3 times. Data reported were means ± standard deviations (SDs). The significance of correlations between Twist1 and clinicopathological characteristics was analyzed by Student's t-test and Pearson's χ^2^ test. Differences between control and test conditions were evaluated by Student's t test or one-way analysis of variance (ANOVA) test using the SPSS 11.5 Statistical Software (version 22.0; SPSS, Chicago, IL, USA). Values of *P* < 0.05 were considered statistically significant.

## SUPPLEMENTARY MATERIALS FIGURE


