# Supplementary material for: Twist1 promotes radioresistance in nasopharyngeal carcinoma
Source: Oncotarget. 2016 Oct 25;7(49):81332–40. doi: 10.18632/oncotarget.12875 (PMC5348396; doi:10.18632/oncotarget.12875)
Supplement: Supplementary file 1 [file oncotarget-07-81332-s001.pdf]

Twist1 promotes radioresistance in nasopharyngeal carcinoma

SUPPLEMENTARY FIGURE

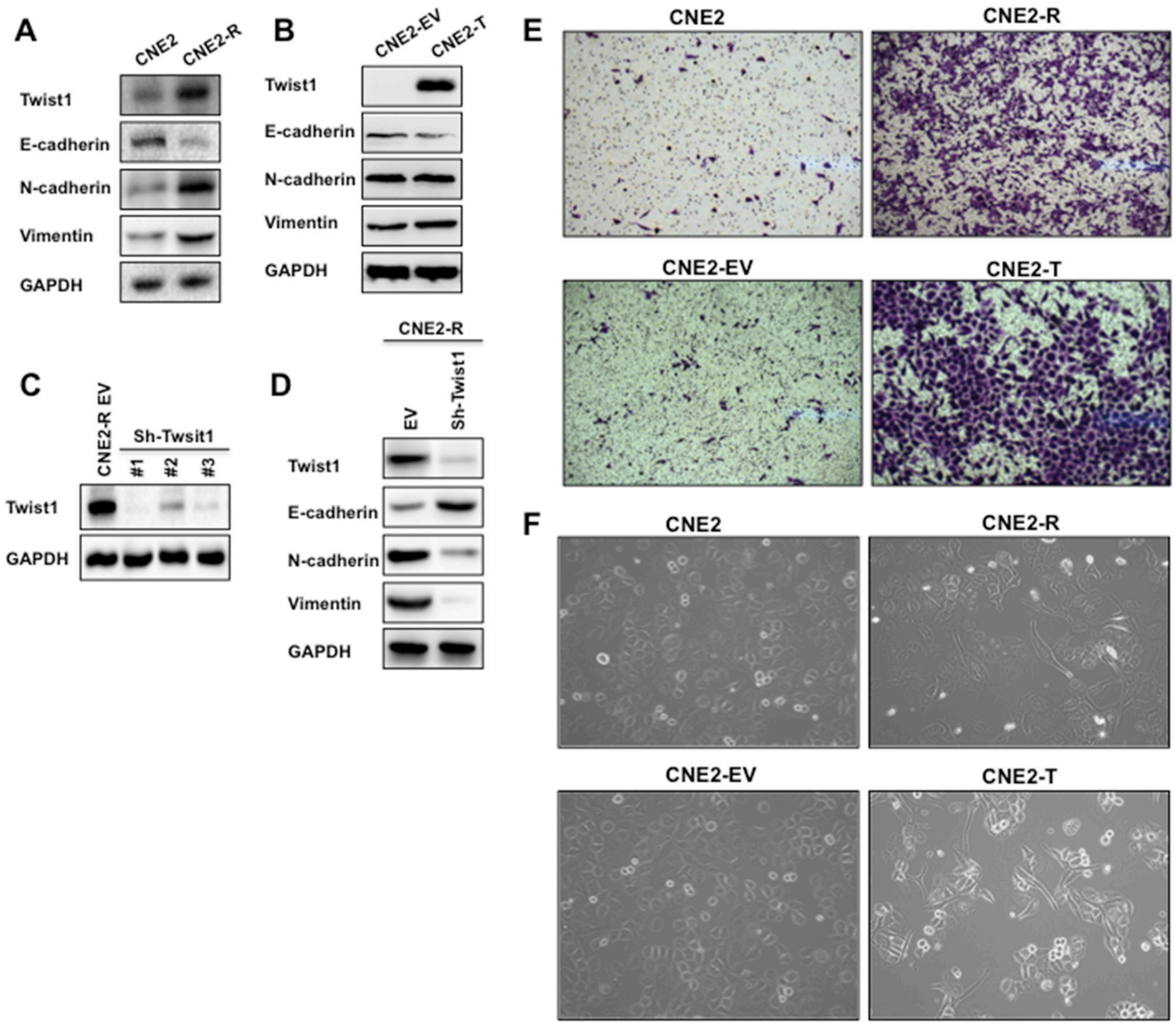

Supplemenary Figure S1: Twist1 induces EMT-like phenotype in NPC cells. A. The IB analysis of WCLs derived from CNE2 and CNE2-R cells specific for Twist1, E-cadherin, N-cadherin, Vimentin. **B.** The IB analysis of WCLs derived from CNE2 and CNE2-T cells. **C.** The IB analysis of WCLs derived from CNE2 cells transfected with Sh-Twist1 lentivirus. **D.** The IB analysis of WCLs derived from CNE2 control and CNE2 Sh-Twist1 cells **E.** The representative images of transwell assay in different cell lines. Photos were taken under 200X magnification. **F.** The representative morphologic images of different cell lines. Photos were taken under 200X magnification.
